# Systemic delivery of P42 peptide: a new weapon to fight Huntington’s disease

**DOI:** 10.1186/s40478-014-0086-x

**Published:** 2014-08-05

**Authors:** Yoan Arribat, Yasmina Talmat-Amar, Alexia Paucard, Pierre Lesport, Nathalie Bonneaud, Caroline Bauer, Nicole Bec, Marie-Laure Parmentier, Lorraine Benigno, Christian Larroque, Patrick Maurel, Florence Maschat

**Affiliations:** Institut de Génomique Fonctionnelle (IGF), CNRS-UMR 5203, Inserm-U661, University Montpellier UM1-UM2, Montpellier, F-34094 France; Medesis Pharma, Baillargues, France; Institut de Recherche en Cancérologie de Montpellier (IRCM), Inserm-U896, University Montpellier1, Institut régional du Cancer de Montpellier, Montpellier, F-34298 France

**Keywords:** Huntington’s disease, Mouse model, Peptide, Microemulsion

## Abstract

**Background:**

In Huntington’s disease (HD), the ratio between normal and mutant Huntingtin (polyQ-hHtt) is crucial in the onset and progression of the disease. As a result, addition of normal Htt was shown to improve polyQ-hHtt-induced defects. Therefore, we recently identified, within human Htt, a 23aa peptide (P42) that prevents aggregation and polyQ-hHtt-induced phenotypes in HD *Drosophila* model. In this report, we evaluated the therapeutic potential of P42 in a mammalian model of the disease, R6/2 mice.

**Results:**

To this end, we developed an original strategy for P42 delivery, combining the properties of the cell penetrating peptide TAT from HIV with a nanostructure-based drug delivery system (Aonys® technology), to form a water-in-oil microemulsion (referred to as NP42T) allowing non-invasive *per mucosal* buccal/rectal administration of P42. Using MALDI Imaging Mass Spectrometry, we verified the correct targeting of NP42T into the brain, after *per mucosal* administration. We then evaluated the effects of NP42T in R6/2 mice. We found that P42 (and/or derivatives) are delivered into the brain and target most of the cells, including the neurons of the striatum. Buccal/rectal daily administrations of NP42T microemulsion allowed a clear improvement of behavioural HD-associated defects (foot-clasping, rotarod and body weights), and of several histological markers (aggregation, astrogliosis or ventricular areas) recorded on brain sections.

**Conclusions:**

These data demonstrate that NP42T presents an unprecedented protective effect, and highlight a new therapeutic strategy for HD, associating an efficient peptide with a powerful delivery technology.

**Electronic supplementary material:**

The online version of this article (doi:10.1186/s40478-014-0086-x) contains supplementary material, which is available to authorized users.

## Introduction

Huntington’s Disease (HD) is a progressive neurodegenerative disorder caused by an abnormal expansion of polyQ (>35) domain in the N-terminus of the Huntingtin protein (Htt) [1]. The worldwide prevalence of HD is 5–10 cases per 100,000 persons. Currently, only symptomatic treatments are available [2]. One of the histopathological marker of HD is intranuclear aggregates formed by the polyQ-mutated Htt protein. These insoluble structures trap crucial proteins [3-5] and can physically block cellular traffic [6-8] and alter for instance the ubiquitin-proteasome degradation process [9,10]. Altogether these defects finally contribute to the neurodegeneration occurring primarily in the striatum and involving other brain areas at later stages of the disease.

Although it is well established that the disease onset occurs as a consequence of expanded polyQ, other domains of Htt could also influence the pathogenicity of polyQ-hHtt. Indeed, the first 17 amino acids (N17 region) and the proline-rich region (PRR region), both flanking the polyQ stretch were found to influence aggregation [11,12].

Most research undertaken to find a cure for HD focus on the mutated allele (trying to reduce its expression) [13], or on the polyQ stretch that is responsible for aggregation, but that is also present in other neurodegenerative diseases [14,15]. Our approach is based on the observation that the ratio between normal and mutant Huntingtin is a crucial factor in the onset and progression of the disease [1,16-22]. In a previous work, we confirmed the protective role of wild-type N-terminus Htt [22]. We further screened human hHtt, for peptides able to prevent polyQ-hHtt aggregation in HeLa cells. To this end, 548aa N-terminal part of hHtt has been fragmented in shorter fragments according to domains lying in the protein. This step-by-step approach allowed the identification of a 23aa peptide (P42), lying in the N-terminus (position 480–502 aa) of normal human Htt (hHtt), able to prevent aggregation *in cellulo* in HeLa cells, and to retain protective properties on different phenotypes induced by the expression of the polyQ-Htt *in vivo* in a *Drosophila* model of HD (on eye degeneration, axonal trafficking and larval locomotion for instance) [8]. P42 is able to prevent eye degeneration of flies expressing hHtt exon1 (covering 67aa, but not P42) with expanded polyQ [8]. Interestingly, this effect of P42 is specific of HD, and not of other polyQ-induced diseases [8]. This showed that P42 is able to act through hHtt 67 aa exon 1, independently to the polyQ stretch.

These previous data suggested that P42 could represent a new therapeutic agent to specifically target HD hallmarks. To test this possibility, we decided to analyse P42 protective properties in another model of HD, preferentially mammalian. In order to better quantify the protective power of P42, we choose to test R6/2 mice that express, as in the fly model, exon1 of human Huntingtin bearing 144 CAG repeats, and which do not contain the P42 domain [23,24]. These mice exhibit early and severe behavioural phenotypes, associated to histological biomarkers mimicking HD. Symptoms start very early at week 5, leading to a premature death in weeks 12–15. In particular, R6/2 mice present neuroanatomical abnormalities including progressive reduction of the brain volume. They are also characterized by the presence of widespread nuclear inclusions of mutant polyQ-Htt in brain neurons. In addition, behavioural abnormalities are also present, including loss of coordination [25].

In order to test P42 in R6/2 mice, we developed a new peptide-based strategy. Peptides have many therapeutic applications, principally due to their efficiency, their selectivity and a high specificity compared to other molecules. Another important advantage concerns their degradation in amino acids that present a low risk of toxicity. Nevertheless, peptides present some limitations, related to their low capability to cross membrane barriers, short half-life, and difficulties of administration. To overcome these problems and optimize the delivery and action of P42, we adapted two complementary and original strategies. First, in order to cross cell membrane barriers efficiently and reach cytoplasmic and nucleic compartments, we fused P42 to the protein transduction domain of TAT from HIV. This 11aa Cell Penetrating Peptide (CPP) can be efficiently used as a vehicle to deliver fused peptides into living cells [26,27], *via* endocytosis [28]. We first tested P42-TAT fusion peptide in HeLa cells for its abilities to transduce cells, and to rescue polyQ-hHtt aggregation. Secondly, in order to optimize the pharmacokinetic characteristics of P42 (serum half life and distribution profile) and to provide a non-invasive route for repetitive delivery of this fusion peptide, we used a novel water-in-oil microemulsion drug delivery vector named Aonys® [29,30]. Aonys® provides a transmucosal (buccal) route of administration and enhances CNS penetration, which we verified by MALDI Imaging Mass Spectrometry.

The Aonys® formulated fusion peptide (named NP42T) represents a new product that combines for the first time the properties of a CPP, with a nanostructure-based drug delivery system. The aim of this study was to evaluate the efficacy of this new formulation of P42, NP42T, in the mammalian R6/2 HD mouse model.

Despite the high aggressiveness and the rapid onset of the phenotypes in the R6/2 model, buccal/rectal administration of NP42T significantly improved motor, histological and molecular phenotypes. These studies allowed to divert the natural properties of a Huntingtin domain P42 towards protection against aggregation and toxicity, which represents a new approach to fight against HD.

## Materials and methods

### Peptide synthesis and formulation

To ensure P42 diffusion, we designed a fusion peptide where P42 was conjugated to the 11aa TAT Cell Penetrating Peptide (CPP). The peptide P42-TAT (AASSGVSTPGSAGHDIITEQPRS-GG-YGRKKRRQRRR) was synthetized and purified by Millegen company (France) with either a TAMRA (Tetra-Methyl-Rhodamine) tag at N-terminal position, or without any tag. NP42T is based on Aonys® drug delivery vector [29,30] where P42-TAT has been incorporated in a water-in-oil microemulsion. The lipid mixture Aonys® was prepared extemporaneously by weighing the lipid constituants. The appropriate amount of the peptide P42 powder (Millegen, purity >95%) was dissolved in MilliQ water. At a well-defined ratio, the P42TAT-Aonys® lipid monophasic microemulsion forms rapidly by short vortex mixing of the lipid mixture with the aqueous peptide solution.

### Cell culture experiments and filter retardation assays

HeLa cells were grown and maintained in DMEM medium (Gibco) supplemented with 10% fetal calf serum and glutamine. HeLa cells were grown in 6 well plates, and pDdest53-GFP-hHtt^171aa^-136Q (0.75 μg) was transfected with JetPei reagent (Qbiogene). Equal amounts of pcDNA-Cherry-P42 or control vector were added for each transfection to level the total amount of plasmid DNA. To test the internalization of exogenous peptide, synthesized TAMRA-P42-TAT was added at the same time as pDest53-GFP-hHtt^171aa^-Q136 transfection, at different concentrations (0, 0.5, 1, 5, 10 or 20 μM). Cells were incubated with plasmid and peptide for 12 h. Then the medium was washed twice with PBS, the cells trypsinized, and grown on a coverslip with new DMEM medium for 6 h. This step was necessary to eliminate the peptide that crystallizes on the well. Eventually, cells were fixed with 4% paraformaldehyde and immunostained for microscopic analysis. To observe the internalization of the peptide TAMRA-P42-TAT, the signal was amplified by anti-TAMRA immunostaining (1:500- Invitrogen). Nuclei were counterstained with DAPI (4′, 6 diamino-2-phenylindole). The pictures were taken with a confocal laser scanning microscope model LSM780 (Carl Zeiss).

For filter retardation assays, protein extracts from HeLa cells were performed according to [31], with the following modifications: After transfection, HeLa cell pellets were treated by DNase and resuspended in 150 μl of 1% SDS and 50 mM DTT in PBS. The samples were boiled for 5 min, and diluted with 150 μl of 1% SDS. For each sample, two aliquots of 150 μl were filtered through a cellulose acetate membrane (0.2 μm pore size, Schleicher & Schuell), using a Bio-Rad dot-blot filtration unit. Membranes were then immunodetected with anti-GFP primary antibody (rabbit polyclonal (1:6000) Invitrogen), as indicated. The membranes have been secondary detected by HRP conjugated anti-rabbit antibody (1:50000-Jackson). Immunoreactive spots were detected with Immuno-Star WesternC kit (Bio-Rad), and quantifications have been performed with Image Lab3.0 software.

### Pharmacokinetics

To test the stability of the peptide in the brain, single intracerebroventricular (ICV) injection of peptides was performed in the right ventricle of C57BL/6 mice as described in [15]. 5 μg or 1 μg of TAMRA-P42-TAT were stereotaxically injected into the right lateral ventricle (coordinates 1 mm right lateral to bregma and 3 mm ventral to the skull surface) of C57BL/6 J mice using a 10 μl Hamilton syringe at a rate of 0.2 μl to 1 μl/min. As a control, saline was injected in the same volume and at the same rate. Mice were sacrificed at 6 h or 24 h by transcardiac perfusion of PFA.

For MALDI analysis, wild-type C57BL/6 J mice have been injected either by ICV (2.1 μg), or treated with empty NP, or with NP42T (210 μg) microemulsions. For NP and NP42T, four buccal and four rectal administrations (1 ml/kg) have been performed, spaced by 30 minutes to ensure mucosa internalization. Two mice have been treated for each condition and sacrificed 3 h after the injections.

### Mass spectrometry and MALDI Imaging Mass spectrometry (IMS)

MALDI MS and MS/MS analysis: Spectra acquisition has been performed using the 4800 Plus MALDI TOF/TOF^TM^ mass spectrometer (ABSciex) controlled by the 4000 Series Explorer^TM^. MS spectra were acquired in a reflector positive mode (mass range: m/z 700–4000, laser intensity set at 50% and 3000 total laser shots per spectrum, MS resolution ≥ 12000–17000). MS/MS precursor was selected manually with an absolute mass window of 1 Da and each MS/MS spectrum is the result of 1000 total laser shots with a laser intensity set at 50% and a MS/MS resolution ≥ 2000–5000. The fragmentation was performed at collision energy of 2 kV and a collision air pressure set at 10^−6^ Torr.

Tissue preparation and MALDI imaging analysis: Frozen brain sections were cut in 15 μm slices using an HM 550 OVPD cryostat (Fisher Scientific, Illkirch, France), set at −20°C, mounted on ITO coated conductive glass slides and allowed to thaw and desiccate in a vacuum desiccator. Tissue sections were then coated manually with a 10 mg/ml α-cyano-4-hydroxycinnamic acid dissolved in 50% acetonitrile (ACN) and 0.1% trifluoroacetic acid (TFA).

MALDI imaging was performed using the 4800 Plus MALDI TOF/TOF^TM^ Analyzer (AB Sciex). Image acquisition was achieved using the 4800 imaging tool software (MSI imaging). Imaging of brain sections was performed in a reflector positive mode, in the mass range of m/z 500–4000, with a resolution of 50 μm in a 100 × 100 μm raster, and laser intensity set at 80% of full laser intensity as selected within the 4000 Series Explorer^TM^. At each position of the tissue section, an averaged mass spectrum is generated from 1000 consecutive laser shots.

The two dimensional ion image of the tissue section was analyzed using Tissue View^TM^ software (Applied Biosystems/MDS analytical technologies). A colour scale representing signal intensity is exported from Tissue View^TM^ software to provide semi quantitative information.

### R6/2 transgenic and wild-type mice

R6/2 mouse model, based on the expression of the polyQ expanded human exon1 is a well-characterized model showing early and strong symptoms and leading to a premature death at 3 months of age [23-25].

Initially, heterozygous R6/2 males were obtained from Jackson Laboratory (stock 006494). The crossing of these R6/2 males with C57BL/6-CBA F1 females generated the animals used in this study. The pups were weaned at week 3. After the weaning, the pups were housed in groups of mixed genotypes, and data were recorded for each mouse. Females issued from this crossing were treated from week 2 to week 11. Until the appearance of the tremor or clasping behaviour, transgenic mice could not be distinguished from normal mice in their home cage. The experimenters were blind to the genotype of the mice until the end of the protocol. The mice were housed fifteen per cage under standard conditions with ad libitum access to water and food. At the end of the protocol, mice were genotyped by PCR using tail-tip DNA as described [23]. A real time PCR was performed with the following primers *HDexon1 Forward : 5′- CGG CTG AGG CAG CAG CGG CTG T-3′,* and *HDexon1-Reverse : 5′- GCA GCA GCA GCA GCA ACA GCC GCC ACC GCC3′*. At week 11, mice were sacrificed and tissues fixed for histology by transcardiac perfusion of 4% PFA in PBS.

### P42 treatment

Procedures for the care and treatment of animals were carried out according to CNRS guidelines, and the experimental protocol was approved by the institutional animal care committee of the Institut de Génomique Fonctionnelle, in accordance with directives of the French Ministry of Agriculture (agreement number: D34-172-13).

For animal experimentation, a cohort of 30 females was divided into four groups (WT treated with empty NP (n = 5); WT treated with NP42T (n = 8); R6/2 treated with empty NP (n = 8); R6/2 treated with NP42T (n = 9)). Administrations were performed five days a week, at the same time of the day, starting in 2-week-old mice. The treatment was performed until the mice were sacrificed (at week 11). NP42T peptide (600 μg/ml/kg) was administrated twice a day *per mucosa*, with two buccal injections until the mice were 4 week old, and *via* rectal and buccal injections for older animals older.

### Behavioural tests of mice

Body weights of the R6/2 transgenic and wild-type mice were recorded weekly at the same time of the day, from the week 2 [14].

Motor performance was assessed using an accelerating rotarod (Stoelting, Ugo Basile, Biological Research apparatus, Varese, Italy), at weeks 6, 8, and 10 [14]. On the first day, the mice were trained with a trial at an accelerating velocity from 4.5 to 40 rpm. Subsequently, two trials were performed during two consecutive days. In each trial, mice were placed onto the rotarod at a constant speed of 4.5 rpm for 5 seconds, which then accelerated at a constant rate up until 40 rpm, for a maximum of 5 min. The daily trial was composed of two sessions of 5 min separated by 20 min. The latency to fall from the rotarod was recorded every day for each mouse, and the average of two trials was used for statistical analysis.

For the clasping test, mice were suspended by the tail for 30 sec. The frequency and the duration of the foot-clasping posture were scored twice a week [32]. The average of two trials was used for statistical analysis. The tests were performed at 7, 9, and 11 weeks of age.

### Statistical tests

For tests with a single time point of data acquired, either an unpaired *t-*test or one way ANOVA, followed by Fisher’s LSD *post hoc* test was used, when necessary. Otherwise two-way ANOVA ordinary with Time and Group or Genotype and Treatment as between-subject factors was applied, followed by Bonferroni’s *post-hoc* analysis for multiple comparisons. In all cases, significance threshold was set at *p < 0.05*. Prism 6.0 software was used to perform statistical analyses.

### Immunohistochemistry

Coronal brain sections of 50 μm thickness were cut with an electronic microtome (Microm HM 650 V). The slides from bregma −0.60 to bregma −0.80 mm were used for Hematoxylin/eosin staining and immunostained with monoclonal antibody anti-Huntingtin (MAB5374 – Millipore – 1:1000) or polyclonal anti-GFAP (AB5804 – Millipore – 1:1000 [33]). For each animal, systematic pictures were taken with the slide scanner Hamamatsu nanozoomer. The areas of lateral ventricles and cortex thickness were measured with NDP software (Nanozoomer Digital Pathology Virtual SlideViewer). Quantifications of aggregates number and size, or GFAP signal, were scored with ImageJ software from 256 μm × 256 μm images. Representative pictures for these immunostainings obtained with the confocal laser scanning microscope model LSM780 (Carl Zeiss) are shown.

TAMRA-P42-TAT injected mice were also immunostained with rabbit monoclonal anti-DARPP32 (MAb2306 - Cell Signaling Technology - 1: 1600), secondary detected by Alexa 488 anti-rabbit (1: 2000) to label the striatum, and with mouse monoclonal anti-NeuN (MAB377 – Millipore - 1: 1000), secondary detected by Dye light blue anti-mouse (1: 500), as a neuronal marker.

## Results

### Construction of NP42T, a new tool against HD

#### Intracellular delivery of P42 peptide when conjugated with TAT

**Figure 1:**
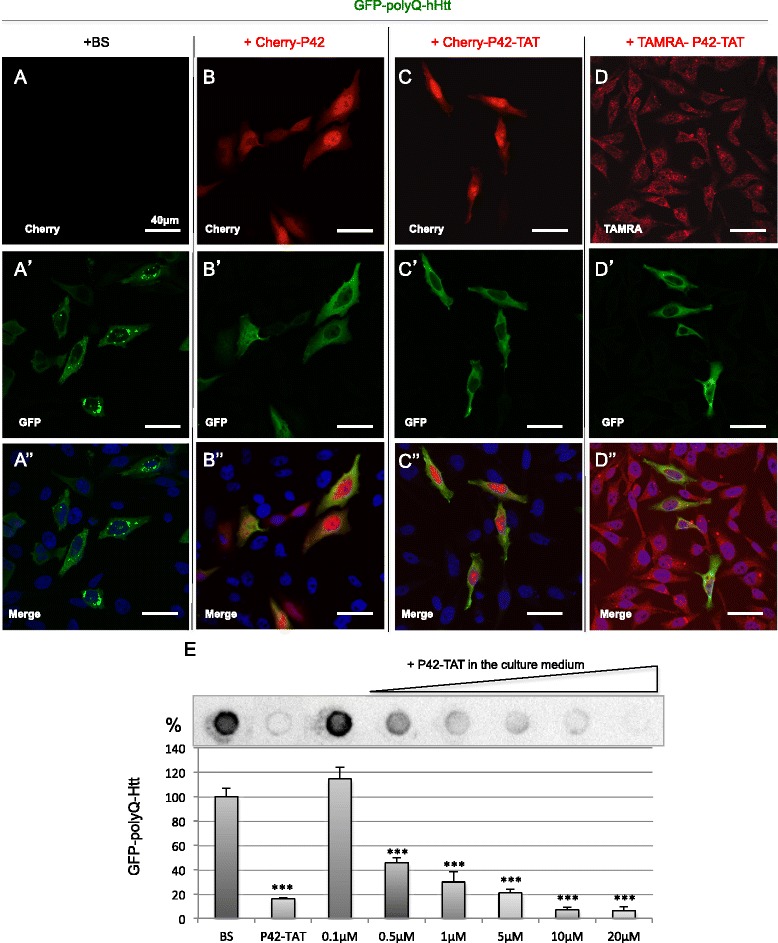
**P42-TAT inhibits efficiently polyQ-hHtt aggregation.** HeLa cells were transfected with GFP-138Q-hHtt^171aa^ (detected in green), in the presence of: BS empty vector **(A, A’, A”)**; Cherry-P42 **(B, B’, B”)**; Cherry-P42-TAT **(C, C’, C”)**; or TAMRA-P42-TAT **(D, D’, D”)**. Cherry and TAMRA are detected in red. As Cherry-P42 **(B)**, Cherry-P42-TAT **(C)** is present both in the cytoplasm and in the nucleus (visualised by DAPI, in blue). The synthetic peptide TAMRA-P42-TAT provided in the culture medium is internalized into the cells and form vesicles **(D)**. The delivery of P42-TAT inhibits aggregation **(C’, D’)**, as does P42 **(B’)**, to compare to A’. **(E)** Filter retardation assays were performed on transfected cells in presence of increasing amounts of P42-TAT (0.1, 1.5, 1, 5, 10 or 20 μM) delivered into the medium. A representative dot blot, where GFP is monitored, is shown. Quantification was performed by Image J on 3 independent experiments and is reported on a graph, with respect to the control (BS) set up at 100%. As a control, quantification of cells co-transfected by GFP-138Q-hHtt^171aa^ and Cherry-P42-TAT is shown (P42-TAT). Data represent means +/−SEM, and were analysed using Student’s *t*. test (*** p < 0,001).

In order to ensure P42 diffusion, we designed a fusion peptide encompassing P42 conjugated to the 11aa TAT Cell Penetrating Peptide (CPP). TAT-conjugated peptides can cross the blood–brain barrier and plasma membranes [28,34]. We first validated the effectiveness of the P42-TAT peptide on polyQ-hHtt aggregation. To this end, HeLa cells were simultaneously transfected with vectors expressing GFP-hHtt^171aa^-Q136 and different forms of the peptide: Cherry-P42 (Figure 1B-B”) or Cherry-P42-TAT (Figure 1C-C”), compared to GFP-hHtt^171aa^-Q136 aggregation in presence of empty vector (Figure 1A-A”). P42-TAT is able to completely rescue the GFP-Htt^171aa^-Q136 aggregation, as did P42 alone (Figure 1B’, C’). We further tested the ability of exogenous P42-TAT to transduce HeLa cells when provided into the culture medium. To this end, we used a synthetic TAMRA end-labelled version of P42-TAT to follow its entry into cultured cells (Figure 1D-D”). As shown on Figure 1D, TAMRA-P42-TAT was taken up by almost all the cells and was concentrated into small vesicles. These data confirmed the high efficiency of transduction of the fusion peptide. We then investigated the ability of TAMRA-P42-TAT to prevent aggregate formation, when provided into the culture medium (Figure 1E). Increasing concentrations of TAMRA-P42-TAT synthetic peptide (from 0.1 μM to 20 μM) drove a clear dose–response effect with a complete inhibition of aggregation in presence of 10 μM peptide. Note that even a 20-fold excess of this protective dose only produced 25% mortality, as assessed by the MTT test [35], while the IC_50_ value could not be measured (data not shown). Altogether these data confirm the protective activity of the P42 peptide even in the presence of TAT.

#### Pharmacokinetics and targeting of P42-TAT into the brain

One challenge to use P42-TAT at therapeutic ends was to find a non-invasive route of delivery, such as buccal administration and to improve P42-TAT bioavailability and uptake into the brain. To this end, we developed a nanostructure-based drug delivery system (Aonys® technology), to form a water-in-oil microemulsion (referred to as NP42T) allowing *per mucosal* buccal/rectal administration, and compared this mode of delivery to intra-cerebroventricular (ICV) injection.

**Figure 2:**
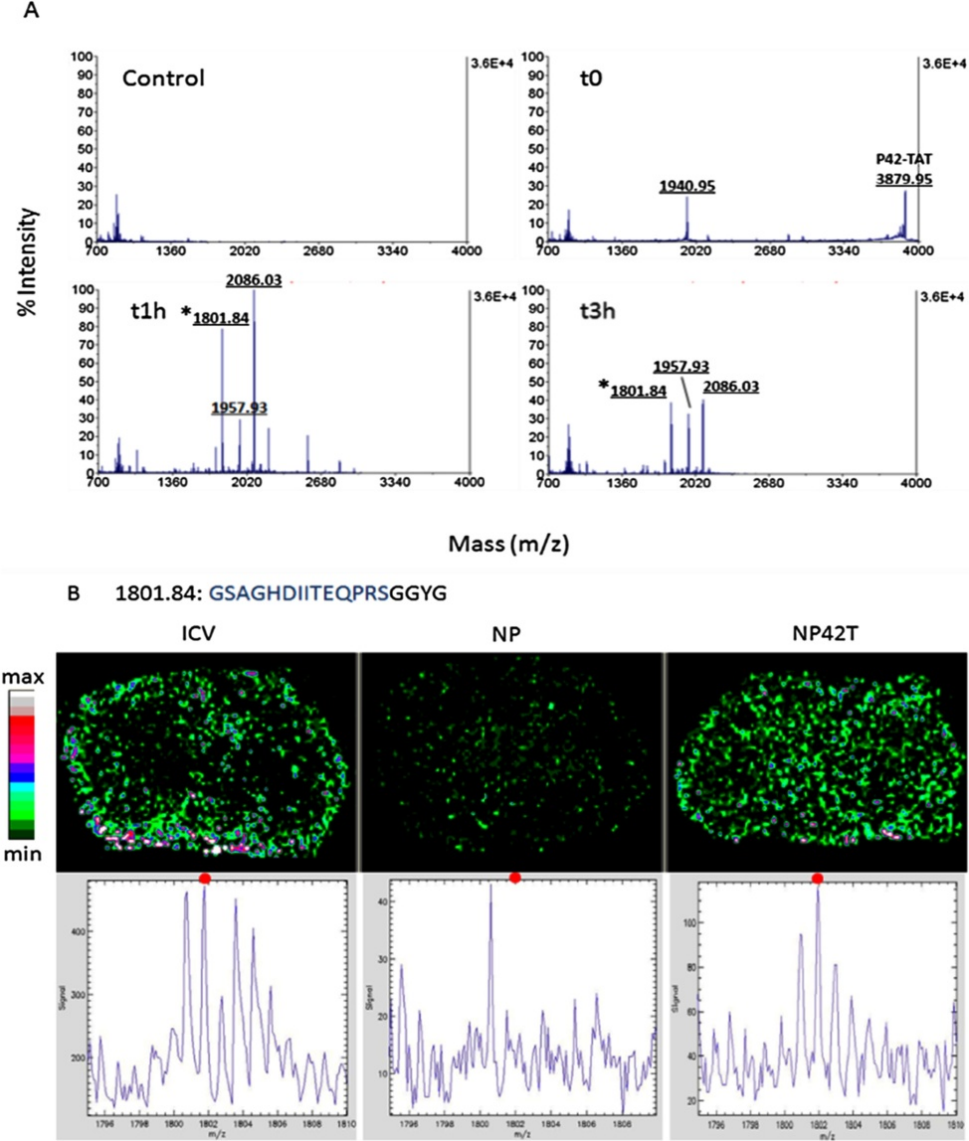
**Pharmacokinetics of P42-TAT in the brain. A**- MALDI-MS analysis (ranging from 699 to 4013 m/z) of mice brain soluble extract before (control), immediately (t0), or after 1 hour (t1h) and 3 hours (t3h) of incubation with 15 μg of P42-TAT peptide. Entire P42-TAT corresponds to m/z 3879.95 at t0. A m/z 1801.84 derivative is detected at t1h and t3h (*) **B**- The distribution of 30 P42-TAT putative derivatives selected have been analysed after IMS and TissueView processing from wild-type mice injected by ICV, or treated with empty NP, or with NP42T microemulsions, as indicated. This allowed to identify one major product, visualized on the brain section image and on the graph (ranging from 1796 to 1808 m/z). Red dot corresponds to the m/z 1801.84 derivative of P42, suggesting that this product is still present in the brain, 3 hrs after the Aonys® administration.

We first analysed P42-TAT degradation in brain extracts at different times of incubation (0, 1, 3 and 6 h) by mass spectrometry. We used MALDI technique to analyse the composition and structure of biological molecules present in solution. In these conditions, full length P42-TAT was degraded within 1 hour, and shorter peptides were detectable by mass spectrometry at 3 hours (Figure 2A). The mass of these products was compared with that of peptides generated by “*in silico*” degradation and 30 masses corresponding to potential peptides were issued from the original P42-TAT.

We hypothesized that some of these degradation products could be present in the brain in addition to the parent P42-TAT peptide. Thus, we analysed the fate of P42-TAT when administered into the brain of wild-type C57BL/6 J mice by ICV injection or by buccal/rectal administration of NP42T encapsulated into Aonys® microemulsion. Since the brain corresponds to approximately 1% of the mouse total weight, we used 100 times more NP42T than for ICV injection.

We used MALDI Imaging Mass Spectrometry [36], whereby the different compounds present in brain sections can be detected simultaneously without further labelling. Using this technology, intact P42-TAT peptide and its degradation products were sought into mouse brain slices three hours after injection. An analysis of the distribution of 30 putative peptide derivatives was performed on brain sections of treated mice. This extensive analysis allowed identifying P42-TAT derivatives only detected in P42-TAT treated mice (Figure 2B). For instance one peptide (m/z 1801.84) is detected in brain extracts after 1 or 3 hours incubation (Figure 2A). A MS/MS characterisation of this peptide confirmed that it corresponds to a part of injected P42-TAT: −GSAGHDIITEQPRSGGYG (Additional file 1: Figure S1).

These data confirm that P42-TAT is able to reach the brain when administrated *via* Aonys® microemulsion.

#### Targeting of the peptide in neuronal cells of the striatum

**Figure 3:**
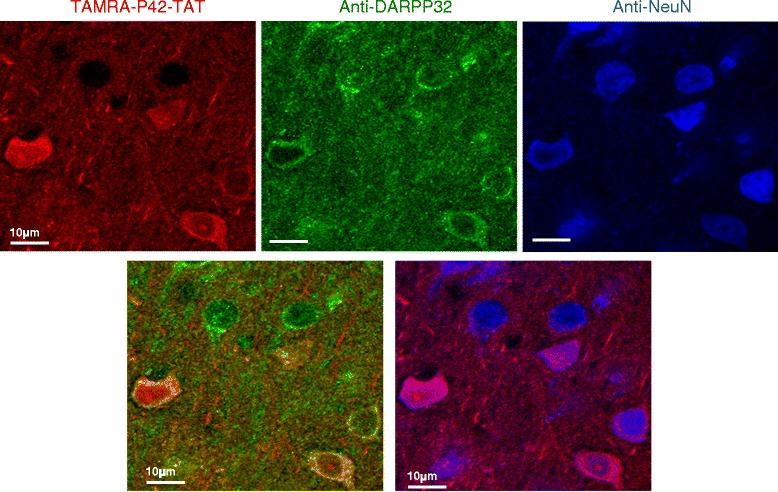
**TAMRA-P42-TAT distribution in the striatum.** TAMRA-P42-TAT (in red) was injected by ICV in wild-type mice, and is detectable in the striatum in DARPP32 positive cells (in green). These cells are also NeuN positive neurons (in blue). Note that TAMRA-P42-TAT is visualized both in the nucleus and the cytoplasm.

To ensure its function and effectiveness *in vivo*, it was important to test the distribution of P42-TAT. To this end, we tested the spreading of a TAMRA-P42-TAT peptide in brains of wild-type C57BL/6 J mice, administrated by intra-cerebroventricular (ICV) injection. Mice were sacrificed 3 hours after injection, and brain sections analysed. On injected-brain sections, vesicles containing the peptide were detectable within the cells and in the ipsilateral and controlateral brain areas from the injection site, including the cortex and the striatum (Additional file 1: Figure S2). We identified that in the striatum, TAMRA-P42-TAT was largely diffusing within the neurons, including medium-size spiny neurons (DARPP32 positive cells on Figure 3). The peptide could be visualized in the different cellular compartments, nucleus and cytoplasm. Whereas P42-TAT could be detected in different neuronal populations (NeuN cells Figure 3) or cilia cells (Additional file 1: Figure S2), but also in astrocytes (data not shown), we could not identify a preferential targeting.

Altogether these data showed that P42-TAT has the ability to diffuse into the brain in the different cell layers, including the striatum. It is also able to reach the different subcellular structures (nucleus and cytoplasm) of the neurons. We also showed that delivering the peptide using Aonys® water-in-oil microemulsion *via* buccal/rectal mucosa guarantees the peptide to reach different parts of the brain, as efficiently as by intracerebroventricular injection.

### Treatment of R6/2 mice by NP42T

In order to confirm *in vivo* the efficiency of P42 combination with TAT and the Aonys® microemulsion, we tested the effect of NP42T in R6/2 mouse model of HD. R6/2 mice are a particularly suitable model for two reasons: first, P42 protective properties have been tested in a *Drosophila* model, expressing exon1 [8], and it was important to test this peptide on a mammalian equivalent model. Second, in R6/2 mice, the P42 domain is lacking, allowing to precisely seizing the protective properties of exogenously administered P42.

R6/2 mice suffer from a progressive decrease in body weight and resting tremor, movements described as resembling chorea [23,24]. Administrations of P42-TAT/Aonys® (NP42T) or empty (NP) microemulsions *via* buccal and/or rectal mucosa were performed daily, 5 days *per* week, in R6/2 and in wild-type C57BL/6 J mice. NP42T was administrated between week 2 and week 11: twice orally during the first two weeks and orally *plus* rectally from week 4 to week 11. Several polyQ-hHtt behavioural associated defects were analysed (foot-clasping, rotarod or body weights), and several markers were evaluated on brain sections (size of ventricles, aggregation and astrogliosis).

#### NP42T reduces behavioural defects of R6/2 mice

To evaluate the therapeutic potential of P42-TAT, we analysed different behaviours.

**Figure 4:**
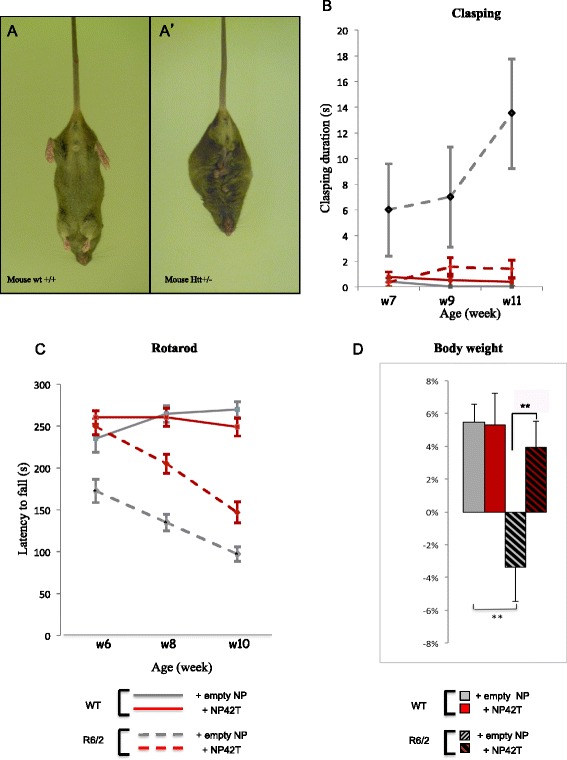
**Aonys® NP42T treatment ameliorates behavioural deficits in R6/2 mice. (A)** R6/2 mice present a foot-clasping behaviour **(A’)** absent in WT littermates **(A)**. **(B)**. Only groups treated with NP42T show a clear inhibition of clasping duration at 11wks: Two way ANOVA (F(Genotype x Treatment)_1,25_ = 6.4, p = 0.0179). Post-hoc comparison (Bonferroni’s test): **p < 0.01 WT (NP) *vs* R6/2 (NP); **p < 0.01 R6/2 (NP) *vs* R6/2 (NP42T), with two way ANOVA (F(Group x Time)_6,78_ = 1.0, p = 0.4022) similar over time. **(C)** Rotarod performances of wild-type (WT) or R6/2 mice treated with NP42T or with empty NP (placebo) at 6, 8 and 10 weeks of age. Motor deficit is identified in R6/2 mice already at week6. Beneficial effect is observed for R6/2 treated with NP42T: Two way ANOVA (F(group x time)_6,78_ = 2.362, p < 0.05) indicates that rotarod deficit of the four groups of mice was different over time. Post-hoc comparison (Bonferroni’s test): 6wks *p < 0.05 R6/2 (NP) *vs* R6/2 (NP42T); *p < 0.05; 8wks ***p < 0.001 WT (NP) *vs* R6/2 (NP); *p < 0.05 R6/2 (NP) *vs* R6/2 (NP42T); 10wks ***p < 0.001 WT (NP) *vs* R6/2 (NP); ns R6/2 (NP) *vs* R6/2 (NP42T). **(D)** Body weight of R6/2 mice is recovered by both early and late administrations of NP42T. Body weight variations were measured in WT and R6/2 mice, treated with empty NP (placebo) or with NP42T. Graphs represent the body weight evolution from 8wks to 11wks in percentage. In HD mice, NP42T inverts the curves and restores a gain of weight in pre-symptomatic. One way ANOVA: (F_(3,24)_ = 5.2, p < 0.01). Post-hoc comparison (Fisher’s LSD): **p < 0.01 WT (NP) *vs* R6/2 (NP); **p < 0.01 R6/2 (NP) *vs* R6/2 (NP42T). Data represent means +/−SEM (n = 5-9 per group).

- When suspended by the tail, normal mice spread their four limbs, whereas R6/2 mice clasp their hind- and forelimbs tightly against their thorax and abdomen (Figure 4A, A’). Six-week-old R6/2 animals begin to exhibit this clasping reflex, which can be easily used to examine novel treatments. We first assessed the effect of buccal mucosa administration of NP42T on foot clasping (Figure 4B). The duration of clasping posture over a 30 seconds period increased in placebo-treated R6/2 mice, with a mean score of 13.7 ± 4.27 s in 11 week-old animals. NP42T treatment reduced massively this defect, with a 10 fold better score, showing a clasping duration of 1.35 ± 0.71 s in 11 week-old R6/2 mice (Figure 4B), which was indistinguishable from the wild-type score. Therefore, NP42T treatment clearly prevents hindleg reflex with a durable effect.
- Around the same age, R6/2 mice also begin to display other gradual changes in motor function such as stereotypical hindlimb grooming, and the emergence of some involuntary movements. As a result, their motor coordination progressively deteriorates, which can be detected as a reduction in the time they can stay on a rotating rod, the so-called Rotarod. Thus, using the rotarod test, we analysed motor coordination and balance performance of R6/2 mice treated with NP42T or empty NP (Figure 4C). Rotarod capacities were tested from week 6, when R6/2 mice begin to present motor defects. At that time, R6/2 mice, which had already been treated for 4 weeks with NP42T, presented normal motor performance (249 ± 9.7 s) similarly to wild-type littermates (234.6 ± 15.9 s). On the other hand, placebo-treated R6/2 mice showed significantly lower motor performances (172.5 ± 13.82 s) (Figure 4C). The NP42T early and chronic treatments allowed a delay of motor defects onset in a sustained manner. Ten week-old NP42T-treated R6/2 animals presented a lower score (146.83 ± 12.69 s) compared to wild-type mice treated with empty NP (placebo) or NP42T (respectively 269.75 ± 9.69 s and 248.72 ± 10.59 s), but maintained an important advantage compared to placebo-treated R6/2 mice (97 ± 8.56 s). In the presence of NP42T, motor deficits were delayed by approximately 4 weeks (Figure 4C).

These results show that NP42T is able to significantly rescue behavioural dysfunctions of R6/2 when using chronic administrations.

#### NP42T impacts peripheral phenotypes

The Aonys® formulation technology allows the delivery of the therapeutic peptide NP42T *via* the lymphatic system and general circulation to the central nervous system as well as to other organs. Indeed, peripheral symptoms have an important impact on HD patient survival. Cardiac dysfunctions and diabetic anomalies contribute to HD patient mortality [37]. Among peripheral phenotypes, one of the major defects leads to an alteration of the body weight evolution. Indeed, R6/2 mice begin to lose weight at week 8 (Figure 4D). Between week 8 and 11, wild-type cohorts exhibited a body weight gain of 5.47% ± 1.1. During the same time period, placebo-treated R6/2 animals showed a weight loss of −3.39% ± 2.1, whereas NP42T-treated R6/2 mice exhibited an increase of 3.92% ± 1.61 of their mean body weight. These results showed that NP42T was able to restore the body mass curve (Figure 4D).

#### NP42T reduces histopathological markers of HD in R6/2 mice brains

**Figure 5:**
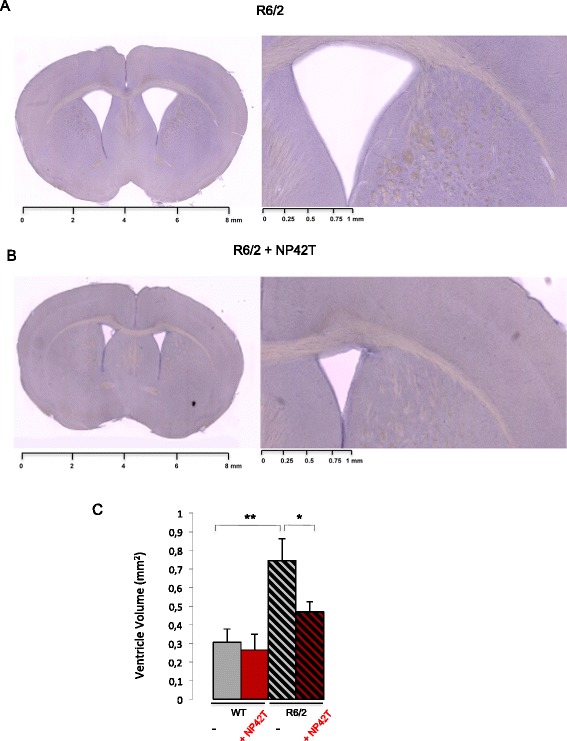
**NP42T delivery reduces ventricle enlargements in R6/2 mice. (A, B)** brain sections of 11wks R6/2 mice treated with empty NP from 9wks to 11wks **(A)**, or with NP42T **(B)** (scale from 0 to 8 mm) with enlargements shown (scale from 0 to 1 mm). Brain sections have been selected with the slide scanner Nanozoomer Hamatsu, and correspond to −0,6 or −0,8 mm bregma point. In presence of NP42T, the ventricle size is closed to WT. **(C)** R6/2 and WT mice were treated with empty NP (−) or with NP42T from 2wks to 11wks. Data represent means +/−SEM (n = 4-8 per group), and were analysed using one way ANOVA: (F_(3,18)_ = 6.4, p < 0.01). Post-hoc comparison (Fisher’s LSD): **p < 0.01 WT (NP) *vs* R6/2 (NP); *p < 0.05 R6/2 (NP) *vs* R6/2 (NP42T).

R6/2 brains are characterized by an atrophy of the neostriatum [24]. When 11 week-old, mice do present an abnormal organisation of the brain, with a clear enlargement of the ventricles (Figure 5A). To investigate whether the NP42T peptide was able to prevent structural changes observed in this HD model, mice brain sections were prepared and lateral ventricle area measured (Figure 5). Ventricle enlargement in R6/2 is at the expense of the striatum area, and of the cortex [38]. At week 11, R6/2 striatum and cortical thickness were only slightly affected compared to wild-type (Rattray et al., [24]) that were not significantly recoved by NP42T (data not shown). A clear rescue was however observed on lateral ventricle enlargement (Figure 5). NP42T-treated R6/2 mice exhibited a mean ventricle area of 0.468 ± 0.06 mm^2^ (Figure 5B), similar to wild-type animals 0.306 ± 0.07 mm^2^ (not shown), whereas placebo-treated R6/2 mice presented a significant enlargement of the ventricles with a mean area of 0.745 ± 0.1 mm^2^ (Figure 5A).

**Figure 6:**
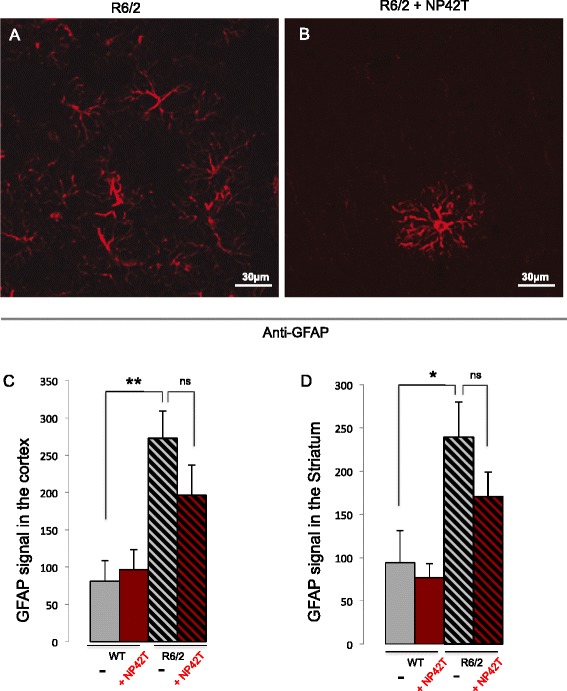
**NP42T delivery reduces astrogliosis in R6/2 mice.** R6/2 mice treated with placebo present an important GFAP positive astrogliosis in the cortex **(A)** and in the striatum (not shown). R6/2 mice treated with NP42T present lower GFAP positive astrogliosis in the cortex **(B)** and in the striatum (not shown). Pictures were obtained by laser confocal microscopy (Zeiss LSM 780). The quantification shows that NP42T reduces astrogliosis in the cortex **(C)** and the striatum **(D)**, but the recovery is not significant. Data represent means +/−SEM (n = 4-7 per group), and were analysed using one way ANOVA: GFAP in the cortex (F_(3,19)_ = 4.9, p < 0.01) and GFAP in the striatum (F_(3,18)_ = 6.1, p < 0.05). Post-hoc comparison (Fisher’s LSD): **p < 0.01 WT (NP) *vs* R6/2 (NP); ns R6/2 (NP) *vs* R6/2 (NP42T) in the cortex and *p < 0.05 WT (NP) *vs* R6/2 (NP); ns R6/2 (NP) *vs* R6/2 (NP42T) in the striatum.

Loss of brain volume in HD is also accompanied by a significant increase in astrocyte number, as shown when using a specific immune-marker GFAP (Figure 6A). We further analysed the astrogliosis process and identified that NP42T lowers the number of astrocytes in the brain cortex (Figure 6B) (with the mean GFAP signal of 196 ± 40 for NP42T-treated mice compared to 272 ± 36 for placebo-treated mice) (Figure 6C) and in the striatum (with the mean GFAP signal of 171 ± 28 in NP42T *versus* 239 ± 41 in placebo-treated R6/2 cohorts (Figure 6D), which correlates with a rescue of the brain volumes in NP42T-treated mice, as compared to placebo treatment (Figure 5).

**Figure 7:**
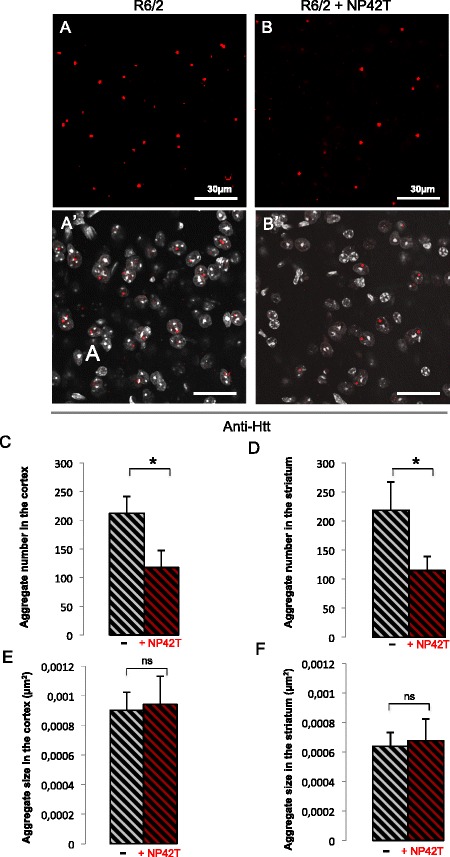
**NP42T reduces the aggregate number in R6/2 model.** R6/2 mice treated with the placebo generate a large number of Huntingtin aggregates in the cortex (in red) **(A, A’)** and in the striatum (not shown). R6/2 mice treated with NP42T present a decreased number of aggregates in the cortex **(B, B’)** and the striatum (not shown). The DAPI staining in grey **(A’, B’)** confirms that the number of cells remains identical. Pictures were obtained by laser confocal microscopy (Zeiss LSM 780). The quantification shows that NP42T reduces the number **(C, D)**, but not the size **(E, F)** of the aggregates in both the cortex **(C, E)** and the striatum **(D, F)**. Data represent means +/−SEM (n = 7 per group), and were analysed using Student’s *t.*test (ns: not significant; ** p < 0.05).

Finally, to determine whether NP42T was able to target the mutant polyQ-hHtt aggregates present in R6/2 mice, as shown in HD *Drosophila* model, we monitored both the number and the size of these nuclear inclusions (Figure 7A-A’, B-B’). After quantification, (Figure 7C-F) we found that aggregates were still present in R6/2 mice treated by NP42T, both in the striatum and the cortex. The number of aggregates was significantly reduced by 50% in both the striatum and the cortex in NP42T-treated R6/2 mice (Figure 7C-D). In contrast, the size of the remaining aggregates remained unchanged in both the cortex and the striatum (Figure 7E-F).

Altogether, these data confirm the powerful protective effect of P42 *in vivo*. The original delivery method based on CPP and a novel microemulsion formulation allowed the peptide to efficiently cross the blood–brain-barrier and its wide spread into the brain, with an efficient rescue on most of the associated HD-phenotypes that were tested.

## Discussion

Despite of recent progresses, HD remains a fatal neurodegenerative disorder. In this context, the identification of P42 represents a new therapeutic avenue. P42 is a 23aa peptide that lays in the N-terminal sequence of Huntingtin. We previously showed that this peptide is able to prevent aggregation of mutant Huntingtin in HeLa cells. Its therapeutic potential was also confirmed *in vivo* in *Drosophila* models of HD, on different phenotypes specifically induced by the expression of polyQ-hHtt [8]. To confirm P42 protective properties, we choose in this report to test P42 in a mammalian animal model.

The use of P42 as a drug in mammals requires the ability to deliver this peptide into the brain. Indeed poor absorption and high enzymatic degradation of peptides are critical for the use of such tools. The need for a chronic treatment requires the development of a non-invasive method of administration, in order to ensure patient compliance. To this end, we joined for the first time two original strategies to provide an efficient distribution of P42 until its targeting into the cells, by using i) Aonys®, a water-in-oil microemulsion [29,30] and ii) a fusion peptide composed of TAT and its cargo P42. We verified that the presence of TAT enhances the diffusion of P42 into a large number of cells. We could also verify that the fusion peptide P42-TAT has kept its protective properties and could diffuse into the cells, targeting the different regions of the brain and the different subcellular compartments. This diffusion of P42-TAT will help to provide better efficacy, preventing aggregate intercellular spreading for instance [39]. In a second step, in order to enhance the low stability and half time of P42-TAT and to enhance its residence time in the CNS, we used the Aonys® microemulsion formulation technology (Medesis-Pharma) to deliver the fusion peptide. P42-TAT was formulated in the water-in-oil microemulsion, allowing *per mucosal* administrations and providing efficient delivery. Therefore, the formulation named NP42T took the advantages of these two delivery technologies. The buccal and rectal administration of such a formulation represents the most appropriate and non-invasive route for chronic peptide delivery. Using this technique, we verified that P42-TAT is targeted to the brain as efficiently as when provided directly by intra-cerebroventricular injection. We also identified that P42 derivatives reside several hours in the brain.

*Per mucosal* administration of NP42T in R6/2 mice allowed early treatment starting at the second week. Thus, we ensured the delivery of the peptide before the first defects appearance. The treatment of R6/2 mice with NP42T improved most of the HD symptoms when administrated pre-symptomatically, delaying for instance the motor impairment observed on rotarod test by around 4 weeks. The benefits observed on the foot clasping phenotypes were also notable until the animals were sacrificed at week 11. For pathological markers, such as aggregation or astrogliosis, the treatment reduced their intensity. Notably, NP42T decreased the number of the aggregates, but not their size. One hypothesis is that by targeting hHtt exon1 [8], P42 might interfere on both the nucleation and aggregation processes, which might prevent new aggregates to form. Note that because of exon1 self-interactions [40], undoing aggregates is more difficult, which might explain that when formed, the size of the aggregates keeps unchanged. However, blocking the formation of new aggregates may allow the neurons to eliminate the preexisting inclusions and the soluble mutant Htt in excess to recover normal functioning, as previously mentioned [41]. Even though the exact role of aggregates and their involvement in cell toxicity remain controversial [42], we observed that the lower number of aggregates is correlated with a recovery of different phenotypes, such as motor performances for instance.

This raises the question concerning the efficiency of P42 for post-symptomatic treatment. In 6 week-old R6/2 mice, most of the symptoms are already developed [24]. In these conditions, a late treatment revealed protective effects on a range of phenotypes, but to a lower extent (our unpublished results). This suggests that P42 might not only target aggregate formation but could also interfere on polyQ-Htt toxicity at other levels, which might delay the progression of the disease.

In humans, the possibility of a pre-symptomatic test, preceded by genetic counseling and carried out in accordance with international recommendations, allows risky subjects to assess their genetic status and to predict whether a subject will develop HD, long before becoming symptomatic [43]. Pre-symptomatic phase is a unique opportunity for early intervention and neuroprotection, in which our peptide could be proposed.

The microemulsion delivery mechanism is likely to mediate a systemic distribution of P42-TAT in the whole organism. Even if the striatum and the cortex are the most affected regions in HD, peripheral symptoms play an important part in the quality of life, and eventually the mortality of HD patients [37]. For instance, cardiovascular defects are responsible for the death in 30% of the patients [44]. For these reasons, whole body delivery of therapeutic molecules seems more appropriate than a local administration (such as viral delivery in a limited region). Indeed, the effect of NP42T on body weight could be explained by more peripheral benefits. We investigated other peripheral phenotypes such as heart mass, heart ventricle size and glycaemia, but no clear variations were observed between wild-type and R6/2 non-treated mice during the time of our study (data not shown). The relatively short timing of the study (from 2 to 11 weeks) and the use of a softer stock of R6/2 mice could explain that we did not notice these peripheral phenotypes previously described in other reports [37]. Nevertheless, the effect of NP42T on body weight evolution might be representative of an action on muscle mass, metabolism, and food intake. Note that no toxicity of P42-TAT has been noticed during this study, since NP42T-treated wild-type mice developed normally, and IC_50_ value on HeLa cell survival (MTT test) could not be measured even in the presence of a large excess of peptide. Moreover the number of neurons, quantified after NeuN-immunostaining, was identical in brains of wild-type mice treated by NP42T or empty NP. Finally neither astrogliosis (detected by GFAP-immunostaining- Figure 6), nor microglia activation (detected by IBA1-immunostaining- data not shown) were identified in NP42T-treated wild-type brains.

The efficient rescue supported by P42 administration represents one of the best therapeutic effects described so far. Therapeutic research currently targets mutant Htt at transcriptional or post-translational level. The recent works in RNA interference or ASO strategies [45] attempt to reduce the expression of the mutant transcript. Despite recent progress, the limitation of this approach lays in the effects on the wild-type allele and the aggravation of the loss of function effects. Biochemical molecules and intrabodies have also been designed against the protein, in order to target abnormal interaction and aggregation processes. In particular, a range of molecules targeting directly the polyQ region of the mutant Huntingtin, have been designed. For instance, the peptide QBP1 and the molecule C2-8 bind the polyQ region and reduce aggregation. These factors drove clear effects in cell models [46,47], but rather mild effects in R6/2 mouse model [14,15]. The combination of TAT with transmucosal microemulsion delivery allowed us to use particularly low doses of P42 (120 ug/week of peptide in adult mice), comparative to what are usually used. In comparison, injection of 500 μg-2 mg/week of QBP1, fused to TAT, had no effect on aggregation process in R6/2 mice, and only a slight effect on body weight [15]. Among the drugs that target the polyQ-hHtt, C2-8 had the ability to also reduce the aggregation process and to improve rotarod performances of R6/2 mice, but this was obtained only in presence of high doses of products (100 or 200 mg/kg/12 h) [14]. The direct targeting of polyQ region might also provide unspecific action, especially if using high doses. Indeed, a wide range of proteins, including transcription factors, physiologically contain a polyQ stretch, which is crucial for their activity, suggesting that chronic treatment might lead to high toxicity. Hence, new strategies that specifically target Huntingtin regions flanking the polyQ have been tested. C4 and V_L_12.3 intrabodies, designed against the N17 region to inhibit nucleation and aggregation, showed encouraging effects in cell model [48,49], but only moderate improvements of R6/2 mice [12]. Hence, the performances of NP42T reported here in R6/2 mice should be greater in other HD models. In particular, P42 effects on lifespan are partial [8], and should be evaluated on less aggressive line.

In summary, P42 combines the specificity of intrabodies targeting flanking regions, but resorts to natural molecular mechanisms normally used by the cells. In addition, the clear dose-effect of P42-TAT that we identified in cell cultures, suggests that the doses that we administered could be enhanced to provide greater benefit.

## Conclusions

To conclude, this report highlights a clear therapeutic potential of P42, a 23aa peptide, localized in endogenous Huntingtin protein [8]. The action of this molecule was potentiated through the combination of transmucosal microemulsion delivery and CPP support. Such a strategy could be applied more generally to other disorders, requiring daily treatments. In early pre-symptomatic treatment, NP42T is protective both on aggregation and on different behavioural or physiological phenotypes. Because HD is a genetic disorder, early screening for patients at risk would enable pre-symptomatic treatment. Later post-symptomatic administration of P42 in R6/2 mice might also help to delay the progression of the disease, but need to be tested further. Nevertheless our data showed that P42 is clearly able to prevent the appearance of symptoms, and to impede the progression of the disease. To enhance the recovery of cell activity, a treatment based on P42 should be combined with other strategies to stimulate autophagic process or proteasome activity for instance [50,51], but also in bitherapies, associating for instance the lithium that was also shown to be protective [30]. Because of the aggressiveness of HD, it seems important to envisage a synergic use of the different therapeutic ways. In this context, P42 might represent one of the most powerful tools to fight efficiently against mutant Huntingtin toxicity.

## Additional file

Additional file 1: Figure S1. MS/MS identification of the m/z 1801.84 peptide. The sequence is confirmed by the detection of numerous b-ions (red arrows) and y-ions (green arrows) generated by the fragmentation of the parent peptide. **Figure S2.** TAMRA-P42-TAT is able to diffuse into C57BL/6 J mice brains. Females were injected into the right ventricle, using ICV injection of 5 μg synthethic peptide. Six hours after the injection, mice were sacrificed and brain sections monitored for TAMRA signal (in red) using laser confocal microscopy Zeiss LSM 780. The peptide fusion is efficiently internalized into the cells surrounding the ventricle (A). Magnification shows that in the cells, TAMRA-P42-TAT is localised in the nucleus and the cytoplasm, and is detected in the ciliary structures (A’). The fusion peptide is able to reach both the ipsilateral and controlateral cortex (B, magnification in B’). TAMRA-P42-TAT is also massively detected in the neurons of the striatum (C) both in the nucleus of the cells, and along the neurites (magnification in C’). The fusion peptide is still detected when mice were sacrificed 24 hrs after ICV injection, and when 1 μg of TAMRA-P42-TAT was injected instead of 5 μg (data not shown). Experiment were reproduced on several animals: n = 12.
