# Supplementary material for: Systemic delivery of P42 peptide: a new weapon to fight Huntington’s disease
Source: Acta Neuropathol Commun. 2014 Aug 5;2:86. doi: 10.1186/s40478-014-0086-x (PMC4149238; doi:10.1186/s40478-014-0086-x)
Supplement: Additional file 1: Figure S1. — MS/MS identification of the m/z 1801.84 peptide. The sequence is confirmed by the detection of numerous b-ions (red arrows) and y-ions (green arrows) generated by the fragmentation of the parent peptide. Figure S2. TAMRA-P42-TAT is able to diffuse into C57BL/6 J mice brains. Females were injected into the right ventricle, using ICV injection of 5 μg synthethic peptide. Six hours after the injection, mice were sacrificed and brain sections monitored for TAMRA signal (in red) using laser confocal microscopy Zeiss LSM 780. The peptide fusion is efficiently internalized into the cells surrounding the ventricle (A). Magnification shows that in the cells, TAMRA-P42-TAT is localised in the nucleus and the cytoplasm, and is detected in the ciliary structures (A’). The fusion peptide is able to reach both the ipsilateral and controlateral cortex (B, magnification in B’). TAMRA-P42-TAT is also massively detected in the neurons of the striatum (C) both in the nucleus of the cells, and along the neurites (magnification in C’). The fusion peptide is still detected when mice were sacrificed 24 hrs after ICV injection, and when 1 μg of TAMRA-P42-TAT was injected instead of 5 μg (data not shown). Experiment were reproduced on several animals: n = 12. [file 40478_2014_86_MOESM1_ESM.pdf]

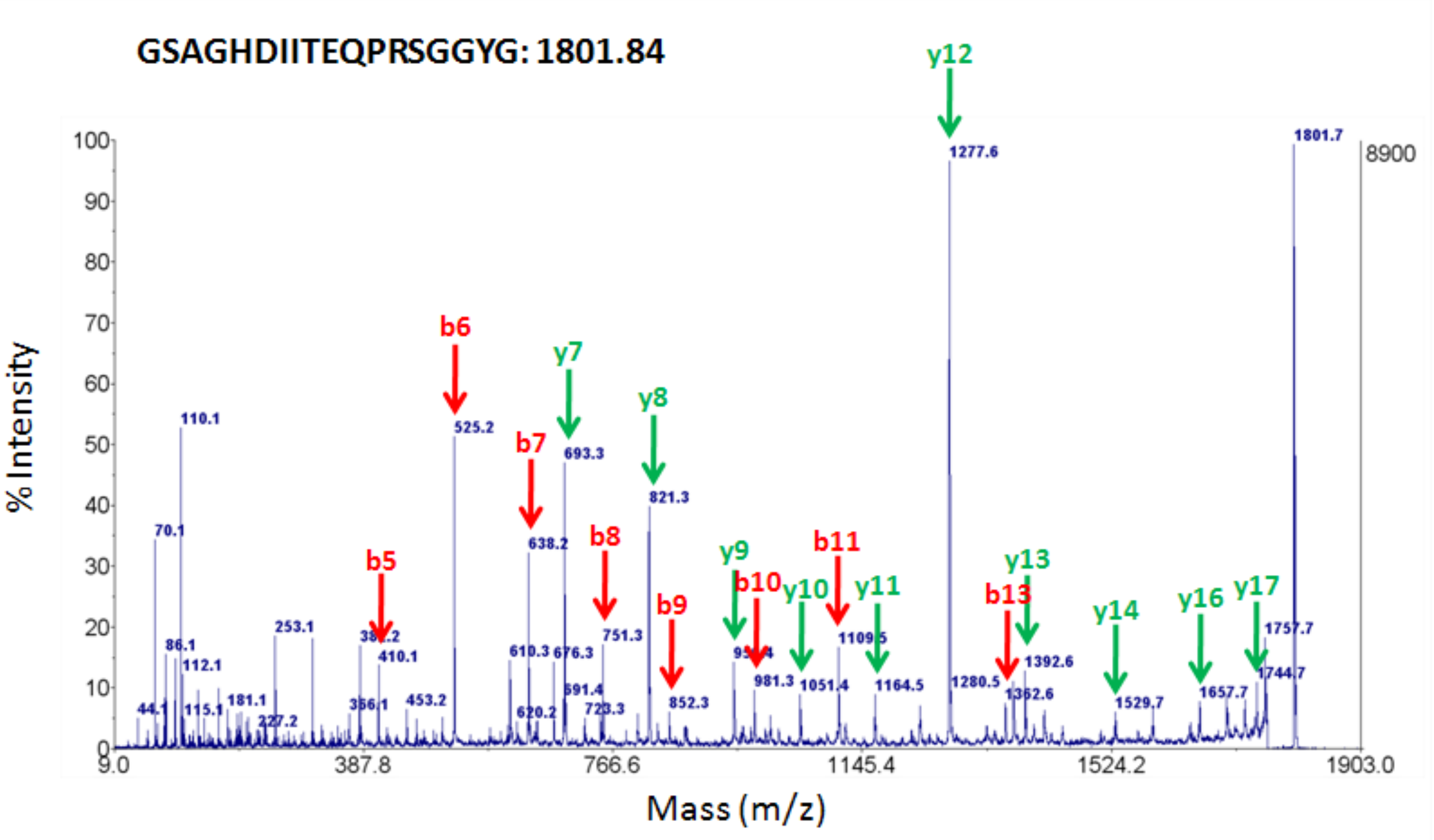

Figure S1- MS/MS identification of the m/z 1801.84 peptide.
The sequence is confirmed by the detection of numerous b-ions (red arrows) and y-ions (green arrows) generated by the fragmentation of the parent peptide.

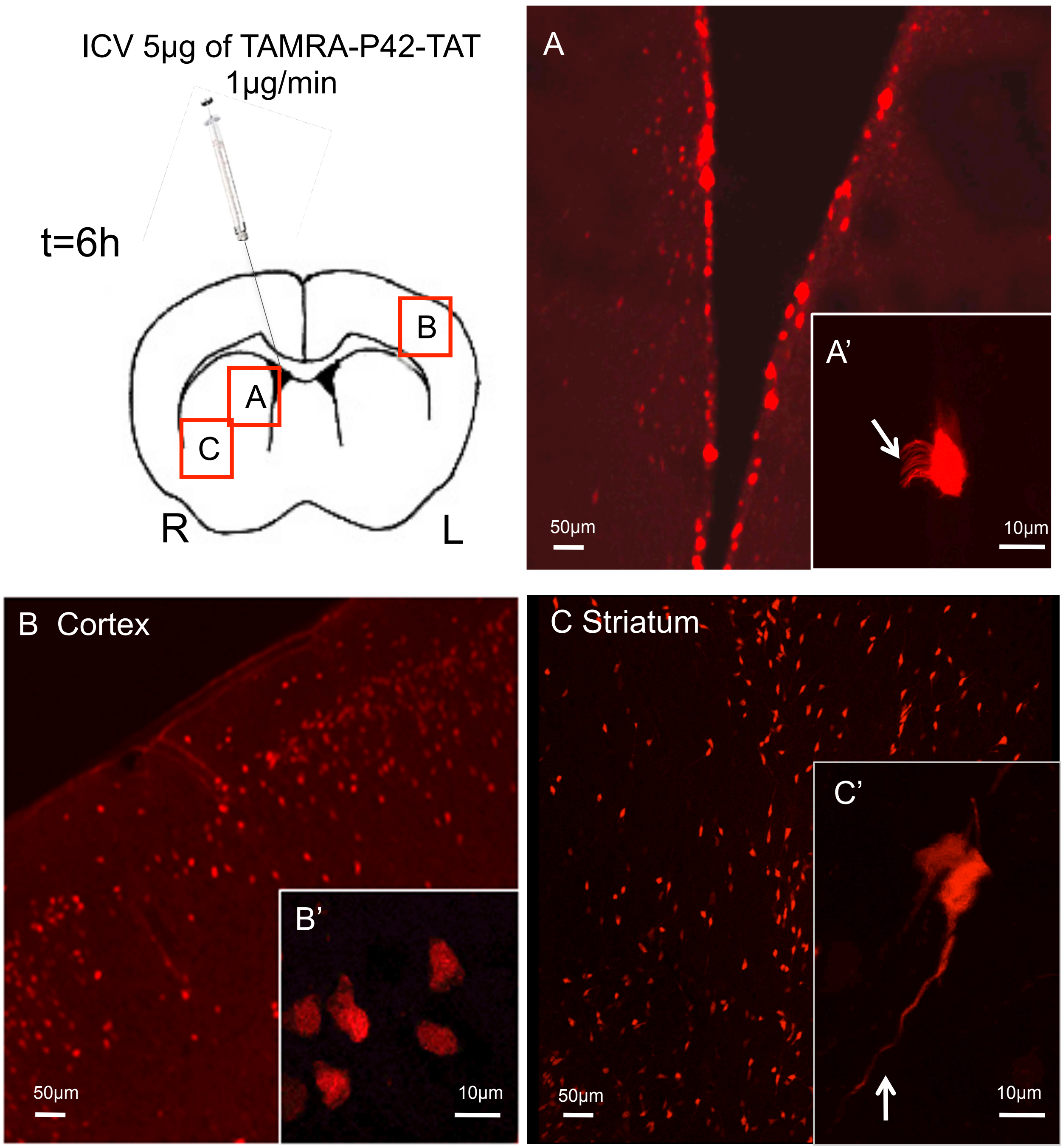

Figure S2: TAMRA-P42-TAT is able to diffuse into C57BL/6J mice brains.

Females were injected into the right ventricle, using ICV injection of 5µg synthetic peptide. Six hours after the injection, mice were sacrificed and brain sections monitored for TAMRA signal (in red) using laser confocal microscopy Zeiss LSM 780. The peptide fusion is efficiently internalized into the cells surrounding the ventricle (A). Magnification shows that in the cells, TAMRA-P42-TAT is localised in the nucleus and the cytoplasm, and is detected in the ciliary structures (A'). The fusion peptide is able to reach both the ipsilateral and controlateral cortex (B, magnification in B'). TAMRA-P42-TAT is also massively detected in the neurons of the striatum (C) both in the nucleus of the cells, and along the neurites (magnification in C'). The fusion peptide is still detected when mice were sacrificed 24hrs after ICV injection, and when 1µg of TAMRA-P42-TAT was injected instead of 5µg (data not shown). Experiments were reproduced on several animals: *n=12*.
